# Sputum microbiome profiling in COPD: beyond singular pathogen detection

**DOI:** 10.1136/thoraxjnl-2019-214168

**Published:** 2020-01-29

**Authors:** Benedikt Ditz, Stephanie Christenson, John Rossen, Chris Brightling, Huib A M Kerstjens, Maarten van den Berge, Alen Faiz

**Affiliations:** 1 Department of Pulmonary Diseases, University Medical Center Groningen, University of Groningen, Groningen, the Netherlands; 2 Groningen Research Institute for Asthma and COPD, University Medical Center Groningen, University of Groningen, Groningen, the Netherlands; 3 Department of Medicine, Division of Pulmonary, Critical Care, Allergy and Sleep Medicine, University of California, San Francisco, the United States; 4 Department of Medical Microbiology and Infection Prevention, University Medical Center, University of Groningen, Groningen, the Netherlands; 5 Institute of Lung Health, University of Leicester, Leicester, UK; 6 Respiratory Bioinformatics and Molecular Biology, University of Technology Sydney, Sydney, New South Wales, Australia

**Keywords:** COPD, sputum microbiome, high-throughput sequencing techniques

## Abstract

Culture-independent microbial sequencing techniques have revealed that the respiratory tract harbours a complex microbiome not detectable by conventional culturing methods. The contribution of the microbiome to chronic obstructive pulmonary disease (COPD) pathobiology and the potential for microbiome-based clinical biomarkers in COPD are still in the early phases of investigation. Sputum is an easily obtainable sample and has provided a wealth of information on COPD pathobiology, and thus has been a preferred sample type for microbiome studies. Although the sputum microbiome likely reflects the respiratory microbiome only in part, there is increasing evidence that microbial community structure and diversity are associated with disease severity and clinical outcomes, both in stable COPD and during the exacerbations. Current evidence has been limited to mainly cross-sectional studies using 16S rRNA gene sequencing, attempting to answer the question ‘who is there?’ Longitudinal studies using standardised protocols are needed to answer outstanding questions including differences between sputum sampling techniques. Further, with advancing technologies, microbiome studies are shifting beyond the examination of the 16S rRNA gene, to include whole metagenome and metatranscriptome sequencing, as well as metabolome characterisation. Despite being technically more challenging, whole-genome profiling and metabolomics can address the questions ‘what can they do?’ and ‘what are they doing?’ This review provides an overview of the basic principles of high-throughput microbiome sequencing techniques, current literature on sputum microbiome profiling in COPD, and a discussion of the associated limitations and future perspectives.

## Introduction

Chronic obstructive pulmonary disease (COPD) is a highly prevalent respiratory disease and the leading cause of morbidity and mortality worldwide.^1 2^ It is heterogeneous, but many patients experience progressive airflow obstruction, exacerbations and/or persistent symptoms of dyspnoea, cough and sputum production.^2^ Exacerbations are heterogeneous and vaguely defined as acute worsening of respiratory symptoms outside of normal day-to-day variation, usually associated with a healthcare utilisation event (eg, prescription of oral steroids and/or antibiotics, emergency room visit). They are associated with accelerated disease progression, increased mortality and major healthcare costs.^2–4^ The heterogeneity of COPD overall, and exacerbations, in particular, complicates the clinical assessment of disease severity and outcome. Determining the fundamental biology underlying COPD heterogeneity is vitally important, as is the identification of biomarkers to guide personalisation of therapeutic strategies.^5–7^

Elucidating the role of respiratory microbiota with respect to COPD pathobiology has potential implications for improving diagnostics and predicting outcomes.^8–12^ The respiratory microbiome, which may be assayed using culture-independent microbial sequencing, is characterised by the micro-organisms and their genomes present in the respiratory tract under specific environmental conditions (eg, exposures or disease states).^13^ Metrics assessing respiratory microbiome composition differ between healthy controls and COPD, and alterations in bacterial abundance and microbial diversity (ie, microbes present in differing proportions) correlate with altered airway inflammatory processes.^14–17^ Current knowledge of the respiratory microbiome still relies mainly on the 16S rRNA gene sequencing approach. Other high-throughput techniques, such as metagenomics, metatranscriptomics and metabolomics, will aid in both a broader investigation of microbial genomes and the examination of associated functional aspects. In this review, we provide an overview of the basic principles of high-throughput techniques for microbiome assessment and analytical approaches. Further, we discuss the current evidence, limitations and future perspectives of sputum microbiome profiling in COPD.

### Basic principles of high-throughput sequencing techniques: 16S rRNA gene sequencing, shotgun metagenomics, metatranscriptomics, metabolomics

Culture-independent high-throughput sequencing techniques have revealed that the human body harbours a microbiome with a complexity far beyond the scope that is detectable by conventional culturing methods.^18 19^ Importantly, cultivation efforts can be extended in order to increase the detection rate of microbes, which represents a significant benchmark for evaluating new culture-independent sequencing technologies.^20^ The choice of technique for microbiome assessment and characterisation should be based on the research question, with consideration of the strengths and weaknesses of each technique. The respiratory tract, certainly in stable COPD, is a low-biomass environment in which the microbial density decreases in a gradient from the upper (URT) to lower respiratory tract (LRT), where microaspiration from the URT is considered to be the dominant source.^21^ Therefore, microbial signals must be discerned from possible contamination during the sample collection and processing prior to analysis.^22 23^ Furthermore, appropriate sample collection and processing following standardised protocols, with particular attention to DNA and RNA extraction methods, should be followed as multiple steps in this process can influence microbe yield and diversity.^24^ A comprehensive discussion of best practices for microbiome analysis overall and with respect to the respiratory tract have been well described previously.^22 23 25 26^

16S rRNA gene sequencing was used in the pioneering work in respiratory microbiome research, and current knowledge still relies mainly on this approach.^8 27^ 16S rRNA sequencing is based on PCR amplification using primers that target the 16S ribosomal gene variable regions of bacterial genomes, which can be used for taxonomic classification.^28^ This approach is rapid, well tested and relatively low cost. By focusing on a specific region of the bacterial genome, it requires only a limited sequencing depth. 16 s rRNA sequencing is used to survey microbial communities, answering the question ‘who is there?’

There are many techniques for analysing microbial communities present within and across samples, but alpha and beta diversity are two of the most common metrics. Alpha diversity captures the number of operational taxonomic units (OTUs) present in a sample (‘richness’) and how they are distributed (‘evenness’), reducing the complexity of these relationships into a single metric. Commonly used alpha diversity metrics include the Shannon index or Simpson index.^26 29^ Beta diversity compares the dissimilarity between samples and provides a matrix of distance. Alpha and beta diversity do not, however, capture the full complexity and dimensionality of microbial community relationships. Thus, more recent work has begun to explore the microbiome in the context of microbial community ecology.^30^ Community ecology theory provides a framework and set of analytical tools to consider how microbes interact with each other and their human host over time and space and in response to exposures or insults (eg, disease states). Newer sequencing approaches that capture microbial function and presence are poised to contribute greatly to our understanding of these ecological dynamics.

16S rRNA gene sequencing exhibits other important shortcomings that may be overcome by newer technologies. For example, it cannot distinguish between live and dead microbes, which may be relevant for several reasons, such as assessing the impact of antibiotic therapy in clinical samples.^31^ Also, 16S rRNA gene sequences are relatively short. Thus, closely related bacteria may share similar sequences, making a separation between different species challenging.^26^ Finally, 16S sequencing analyses are restricted to the detection of bacteria and archaea since viruses or fungi do not carry 16S rRNA genes.

Shotgun metagenomic sequencing is based on unrestricted DNA sequencing of genetic content in a sample. This approach can capture DNA-based viruses and eukaryotic DNA, including fungal microorganisms, in addition to bacteria.^26^ Shotgun metagenomics allows for an unbiased and deeper taxonomical analysis of the microbiome than 16S rRNA gene method, since dissimilar regions of microbial genomes are sequenced. The assembly of short sequencing reads into larger fragments can increase the discriminatory power between microbes, enhancing taxonomic resolution up to the species or strain level.^25 32^ Feigelman *et al* demonstrated how unbiased DNA sequencing in sputum from patients with cystic fibrosis (CF) can enhance in-depth microbiome profiling.^33^ Furthermore, shotgun metagenomic analyses add insight into the question ‘what can these microbes do?’ The potential metabolic capacity of the microbiome can be evaluated by assigning microbial genes to pathway profiles and investigating potential cross-talk between the microbiome and human host and between microbes.^34 35^

Metagenomic sequencing has several weaknesses. As with 16S rRNA gene sequencing, it is unable to distinguish between live and dead microbes. The high sequencing depths necessary for taxonomic classification may be prohibitively expensive, although these costs are diminishing.^25 26^ Most respiratory viruses are RNA-based and therefore not detectable by metagenomic approaches. Additionally, pathway profiling of microbial genes will only allow for the evaluation of potential metabolic capacities, and can thus only conjecture at functional roles.

Metatranscriptomic sequencing examines RNA-level genetic content. RNA sequencing (RNA-seq) provides a survey of microbiota that include RNA-based respiratory viruses.^36–38^ Unlike DNA-based methods, metatranscriptomics focuses on living microbiota. By assessing gene expression (transcriptome) of the microbiota, it can enable a better assessment of microbial functional activity. Further, this approach allows for a simultaneous assessment of the host transcriptome. Thus, metatranscriptomics may provide broader insight into the question ‘what are they doing?’ than metagenomics by allowing for an assessment of functional interactions between different microbiota and between host and microbes.

There are drawbacks to metatranscriptomics as well.^39^ RNA is vulnerable with a short half-life and requires careful collection and storage associated with high costs. Further, results can be biased towards microbial genes with higher transcription rates. Consequently, it is preferable that RNA-based approaches are complemented by DNA-based sequencing so that RNA/DNA ratios and microbial transcriptional activity may be examined. Finally, the application of RNA-seq to microbiome research is still relatively new, especially with respect to the respiratory tract. Therefore, best practices for sample processing and analysis are still evolving.

Metabolomic data generation is based on chromatography techniques and detection methods, such as mass spectrometry or nuclear magnetic resonance, instead of sequencing.^40^ Application of these technologies to microbiome research is still in its infancy and has been better studied in the gut than the respiratory tract. However, the techniques provide an opportunity to more accurately answer the question ‘what are the microbes doing?’ when used in combination with microbial and host sequencing that identify relevant alterations in microbial communities and host response. Microbial-derived metabolites include those synthesised directly by microbes and those produced by the host and biochemically modified by microbes.^41^ They are known to affect host physiology (host–microbiota interactions) as well as dynamics within the microbiome (microbe–microbe interactions). They have been shown to play important roles in health and disease. These include crucial roles in the maintenance of the epithelium and immune cell function, processes dysregulated in COPD pathogenesis.^41 42^ Thus, metabolomics-based analyses provide a promising opportunity for studying microbial-host dynamics within the respiratory tract.

Overall, evolving high-throughput methods are revolutionising microbiome research. Although 16S rRNA gene sequencing is still the most common technology used in respiratory microbiome research, shotgun metagenomics, metatranscriptomics and metabolomics are increasingly applied to microbiome research in other compartments (eg, gastrointestinal tract). A crucial challenge remains the depletion of host DNA, which represents the vast majority of DNA present in low-biomass samples.^22^ Samples can be pretreated to reduce the host genome background, however, its effect on an unbiased microbiome detection remains unknown.^22 43^ Further, standardisation of methods, including sample collection, processing and analytical pipelines, is required in order to further elucidate their utility in respiratory microbiome research.

### Sputum microbiome profiling in COPD: the current body of evidence

The respiratory tract is an anatomically and physiologically complex organ. Physiological parameters, such as pH level, relative humidity or temperature, change along the respiratory tract, creating niche-specific growth conditions for microbes.^44^ Consequently, it is likely that the microbiome varies along the respiratory tract. Furthermore, airway microbiome profiles depend on sampling method (eg, nasal brushing, induced or expectorate sputum, bronchoalveolar lavage (BAL), epithelial brushing, bronchial biopsies) consistent with this niche specificity.^17 45–47^ To exploit respiratory microbiome characteristics for COPD diagnostics, phenotyping and outcome prediction, it is necessary to identify microbiome biomarkers that are easily obtainable in a patient-friendly manner. Sputum closely aligns with these characteristics, making it a preferred sampling technique. Sputum microbiome characteristics, such as microbial diversity or relative abundance, are associated with disease severity and outcomes in stable COPD and exacerbations (see online supplementary table 1).^10 11 45 48 49^

10.1136/thoraxjnl-2019-214168.supp1 Supplementary data

#### Sputum microbiome profiles in stable COPD

In stable COPD, reduced sputum bacterial microbiome diversity has been shown to be associated with more severe airflow obstruction.^17 45 49 50^ Galiana *et al* found that participants with severe airflow obstruction exhibited lower alpha diversity and a higher bacterial load in sputum samples, compared with participants with mild or moderate airflow obstruction.^49^ This finding has since been reproduced in both sputum and BAL sampling.^17 50 51^ These studies also reported a shift in the microbial flora with decreasing lung function towards potentially pathogenic bacterial genera, particularly those belonging to the gammaproteobacteria class (eg, *Pseudomonas* or *Haemophilus* spp). Thus, the observed decrease in microbial diversity appears to be associated with their outgrowth. Although reduced microbial diversity has been identified at other mucosal sites (eg, gastrointestinal tract) in association with chronic inflammatory diseases (eg, inflammatory bowel disease), it remains of interest whether these associations are influenced by therapeutic interventions.^52^ Further, identifying causal relationships between chronic inflammation and microbial community changes is an active area of research.

The association between bacterial load and features of stable COPD appears to be complex and affected by chosen therapies.^49 53^ Garcha *et al* showed that higher bacterial load of potential pathogens, such as *Haemophilus influenzae, Streptococcus pneumoniae, Moraxella catarrhalis*, correlated with more severe airflow limitation in stable COPD.^53^ Further, they reported a positive correlation between bacterial load and inhaled corticosteroids (ICS) dosage. In a randomised controlled trial, Brill *et al* reported that chronic antibiotic therapy, often used as an exacerbation preventative therapy, was associated with a ≥3 fold increase in antibiotic resistance among airway bacteria without decreasing the total bacterial load in sputum.^54^ These associations between therapies and microbial load or function suggest that therapies regularly used in COPD management may have unintended consequences on the microbiome, although the significance of these consequences is unclear.

There is preliminary evidence that sputum microbial communities from COPD patients with more severe airflow obstruction may have shifted such that they lack potentially commensal bacteria, favouring more pathogenic microbes.^49^ Several studies identified potential commensal bacteria in the URT, including *Dolosigranulum* spp and *Corynebacterium* spp, which appear to be associated with respiratory health and pathogen clearance.^55–57^ Galiana *e* *t al* found that the potentially commensal bacterial genus *Actinomyces* was less frequently recovered in sputum from patients with COPD with severe compared with moderate airflow obstruction.^49^ Iwase *et al* showed that commensal respiratory bacteria can directly suppress the outgrowth of potential pathogens belonging to the same genus or family, highlighting the importance of microbe–microbe interactions in maintaining homeostasis.^58^ Potential new therapeutic approaches might not only aim at reducing colonisation of pathogenic microbes but also at increasing colonisation of commensal bacteria in the respiratory tract.

#### Sputum microbiome profiles in COPD exacerbations

GOLD guidelines recommend performing sputum cultures in patients with frequent exacerbations, severe airflow limitation and/or those requiring mechanical ventilation.^2^ While the goal of sputum culture in this setting is to determine the presence of bacterial pathogens and to guide antibiotic treatment, culture is very limited in this role.^59–61^

Evaluating the sputum microbiome with modern high-throughput sequencing technologies might contribute to enhanced exacerbation precipitant diagnosis. Sputum microbiome profiles have been shown to exhibit dynamic changes between stable COPD and exacerbations in at least a patient subset, involving the outgrowth of pathogens, decreased microbial diversity and increased abundance of pathogenic bacterial communities.^11 62^ There is also evidence that bacterial outgrowth, as occurs in overt infection, may not be necessary for bacteria to trigger an exacerbation. Strain shifts towards more pathogenic bacterial strains, without an overall change in bacterial abundance, have been identified as potential precipitants for some exacerbations.^63^

Host biomarkers and microbial profiling in sputum samples suggest that at least three exacerbation subtypes exist, defined by both the airway inflammatory state and the suspected exacerbation precipitant: bacterial, viral and eosinophilic.^10^ Ghebre *et al* found that sputum samples clustered by exacerbation subtype exhibited differing microbial community characteristics.^10^ Exacerbations categorised as bacteria associated had the greatest Proteobacteria abundance and a high Proteobacteria/Firmicutes ratio in their sputum, while exacerbations categorised as eosinophilic exhibited greater sputum microbial alpha diversity.^10^ Mayhew *et al* found that exacerbations classified as bacterial and eosinophilic were more like to be repeated within a subject over time than viral exacerbations.^51^ Thus, exacerbations exhibit substantial biological heterogeneity. Considering exacerbations within the subtyping framework may add considerably to our understanding of underlying mechanisms and provide insight into more appropriate precision-guided management.

Although changes in bacterial community structure appear to occur in only an exacerbation subset, dysbiosis is indeed associated with particular exacerbation features and worse outcomes. Wang *et al* found that the sputum microbial community composition changes significantly from stable to exacerbation state in only ~40% of their cohort.^48^ Participants with microbial dysbiosis had worse health status and lung function, particularly if they also had evidence of eosinophilic inflammation. Furthermore, the abundance of *Moraxella* spp increased in this study, corroborating other reports that find specific pathogenic phyla and genera are enriched in the exacerbation microbiome.^11 62^ Leitao Filho *et al* found that reduced sputum microbial alpha diversity and altered beta diversity during hospitalised exacerbations were associated with increased 1-year mortality.^64^ Interestingly, associations between limited diversity and disease progression has also been described in other lung diseases, such as CF.^65^ Together, these results indicate that the heterogeneous contribution of microbes to exacerbations may be clinically relevant.

Several reports have speculated that the contribution of typically non-pathogenic bacteria to alterations in microbial community ecology may be crucial to exacerbation pathogenesis. Wang *et al* identified an outgrowth of non-pathogenic Proteobacteria, in addition to pathogenic microbiota, during exacerbations. They speculate that this state of dysbiosis, with differing microbe–microbe and microbe–host interactions, leads to a dysregulated host inflammatory response.^11 66^ Huang *et al* reported a positive relationship between the abundance of COPD-associated pathogens during an exacerbation, (eg, *H. influenzae*, *Pseudomonas aeruginosa* or *Moraxella catarrhalis*) and phylogenetically related non-pathogenic bacteria.^62^ Leitao Filho *et al* speculate that the absence of potentially commensal bacterial genera in the sputum microbiome is associated with clinical outcomes in exacerbations.^64^ They found that both the presence of *Staphylococcus* spp (potentially pathogenic) and the absence of *Veillonella* spp (potentially commensal) in sputum were strongly associated with the increased risk of mortality after 1 year. This work highlights the need for a better understanding of the role of both pathogenic and commensal microbes within the respiratory microbiome during exacerbations.

Importantly, two studies did not find changes in diversity metrics between disease stability and exacerbations.^51 67^ Millares *et al* additionally failed to identify changes in bacterial abundance but did report changes in functional metabolic pathways of the microbiome during exacerbations. Mayhew *et al* did not identify a change in diversity metrics but did find an increase in the relative abundance of *Moraxella* spp during exacerbations.^51 67^ These reports provide further evidence of the overall phenotypic heterogeneity in exacerbations, but also emphasise the potential importance of strain shifts without changes in species or class-level microbial community structure in precipitating bacterial exacerbations.

Standardised follow-up studies are required to validate current findings. Eventually, causation and mechanisms between sputum microbial community structure and host inflammatory response require further investigation, which demands causal models and rigorous methods.^68 69^ A promising approach was recently published by Sanna *et al*, using a bidirectional Mendelian randomisation analysis to assess causality between the gut microbiome and metabolic diseases.^70^

#### Sputum microbiome profiles after therapeutic interventions of exacerbations

Current knowledge about the impact of empirical approaches to the treatment of exacerbations on sputum microbiome profiles is still scarce. Wang *et al* reported that steroid treatment alone was associated with reduced alpha diversity, increased abundance of Proteobacteria (including *Haemophilus* and *Moraxella* spp) and an increased Proteobacteria:Firmicutes ratio, whereas opposite trends were found in exacerbations treated with antibiotics (with or without steroids).^11^ Huang *et al* also reported an enrichment of Proteobacteria, in addition to other bacterial phyla with steroid treatment, indicating that this treatment alone might increase the burden of specific microbiota.^62^ In the same study, antibiotic treatment had suppressive effects on this bacterial burden. In contrast to Wang *et al* findings, Huang *et al* observed a trend towards increased microbial diversity after steroid treatment and decreased diversity following antibiotic treatment.

Further research is needed to better understand treatment effects on respiratory microbial dynamics over time as well as microbial–host interactions. Segal *et al* took a promising approach to study the antimicrobial effects of azithromycin in COPD.^71^ Using a combination of 16S sequencing and metabolomics they found decreased alpha diversity in azithromycin-treated BAL samples and increased bacteria-produced metabolites known to influence the host inflammatory response. More mult-‘omic studies integrating microbial and host data are needed to elucidate and characterise the potentially deleterious impact of chronic therapies in COPD to better guide therapy decisions.

In summary, the current body of evidence indicates that the severity of COPD is inversely correlated with sputum microbial diversity and is associated with an outgrowth of potentially pathogenic bacteria. Exacerbation subtypes exhibit altered sputum microbial community characteristics, providing potential targets for precision-guided management. Further studies, integrating microbial and host information, are needed to understand the impact of therapeutic interventions on the microbiome and interactions with the host. Eventually, potential new therapeutic approaches might not only aim at reducing the colonisation of pathogenic microbes but also to either increase the colonisation of commensal bacteria or modulate the associated host response.

### Sputum microbiome profiling in COPD: limitations, challenges and future perspectives

**Figure 1:**
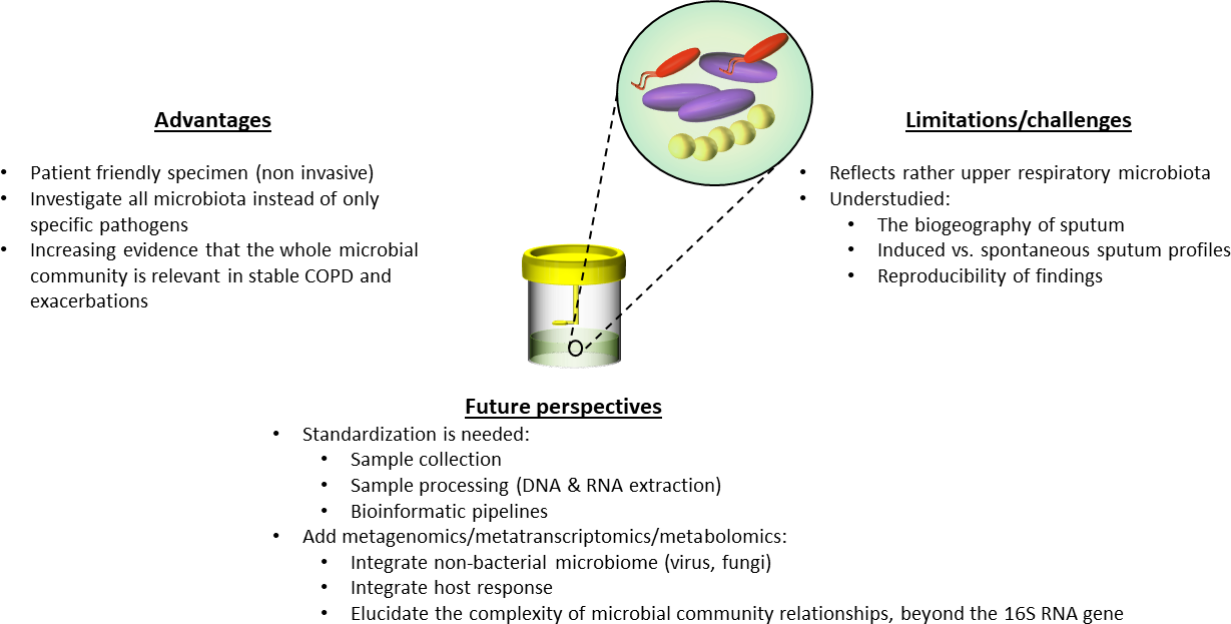
Sputum microbiome profiling—beyond singular pathogen detection. COPD, chronic obstructive pulmonary disease.

There are several limitations of sputum microbiome profiling as well as challenges that should be addressed in future studies to better assess the utility of the technique in COPD microbiome research (figure 1):

#### Limitations and challenges: the biogeography of sputum

LRT sampling methods, such as BAL and bronchial biopsies or brushings, are invasive approaches and thus poorly suited for longitudinal assessment. However, COPD is generally a disease of the LRT. Thus, understanding the similarities and differences between these LRT sampling methods and more easily obtainable sputum sampling is essential for interpretation.^47 72^ In a small study (n=20), Sulaiman *et al* found that induced sputum microbiota were more similar to those obtained by oral wash than those obtained from the lower airway (bronchial biopsies) in participants with and without non-tuberculous mycobacterial infections.^72^ Durack *et al* also reported an enrichment of oral bacteria in induced sputum samples compared with bronchial biopsies.^47^ However, they found that the abundance of genera identified in bronchial biopsies correlated quite well with those detected in both induced sputum and oral wash. Thus, despite the overall difference in microbial composition between URT and LRT sampling, sputum does have utility in assessing LRT microbiome composition, in particular, relative microbial abundance. These correlations between lower and upper airway microbe abundance underscore the theory that microaspiration of microbes from the URT is the primary source of bronchial microbiota.^21 46 73^ While characterising the airway microbiome using sputum should be done considering the limitations, the considerable strengths in comparison to LRT sampling should be considered as well. Sputum is easily obtainable and alterations in the sputum microbiome correlate with clinical outcomes. Thus, sputum microbiome biomarkers may be useful even if they do not accurately reflect the LRT microbiome.

#### Limitations and challenges: induced versus spontaneously expectorated sputum

Differences in microbiome profiles obtained from spontaneous versus induced sputum samples are particularly understudied. Although patients without spontaneous sputum production can safely undergo sputum induction even during exacerbations, preliminary results suggest that microbial composition may be affected by sampling technique.^74–76^ Furthermore, Gershman *et al* found that the duration of sputum induction affects the host cellular and biochemical composition in samples and suggest that the large airways are sampled at the beginning, whereas peripheral airways are sampled at later time periods.^77^ Tangedal *et al* found that microbiota compositions differed between pairwise spontaneous and induced sputum samples in participants with COPD.^76^ Further studies are needed in which protocols, and in particular sputum volume obtained, sample quality and procedure duration, are standardised across expectorated and induced sputum sampling.

#### Limitations and challenges: reproducibility of findings

COPD sputum microbiome studies to date have mainly used a cross-sectional design. Within-individual longitudinal microbial profiling reports are lagging, but data are starting to emerge. Sinha *et al* collected repeated induced sputum samples from four stable patients ith COPD over 9 months. They reported the stability of bacterial composition and diversity over 2 days, with increased variability over 9 months.^78^ In a larger longitudinal cohort (n=101), Mayhew *et al* found relative stability in microbial composition in a participant subset,^51^ while those with more variability were more likely to have higher exacerbation frequency.

Overall, both within-individual and between-cohort reproducibility represent crucial milestones in the development of a biomarker and require further scientific attention and evaluation with respect to sputum microbiome profiles in COPD.^79 80^

#### Future perspectives: integrate the non-bacterial microbiome

16S rRNA sequencing approaches in sputum samples have provided important insights into the dynamics of the bacterial microbiome in stable COPD and exacerbations. However, the respiratory microbiome includes viruses and fungi as well as bacteria. The prevalence and clinical significance of fungi (mycobiome) in the respiratory tract in the context of COPD are particularly understudied.^81 82^ Bacterial-fungal and bacterial-viral interactions can affect microbial dynamics and microbe–host interactions and require further attention.^83 84^ Furthermore, the outgrowth of potentially pathogenic bacteria can be influenced by prior non-bacterial infections. Postviral bacterial respiratory infections are common overall. However, Molyneaux *et* *al* suggest that postviral bacterial dysbiosis is particularly an issue in COPD. They found that rhinovirus infection was followed by an outgrowth of *H. influenzae* in COPD sputum samples and associated with an increased neutrophilic inflammatory response.^85^ They did not observe this outgrowth in healthy controls.

Overall, mechanisms of postviral secondary bacterial infections are known to be complex, mediated by interactions between viruses and bacteria (intermicrobial interactions) as well as the host immune system.^86^ It will be crucial to evaluate not only bacterial microbiome dynamics but viral and fungal dynamics as well, to evaluate the influence of inter-microbial interactions on both homoeostasis and pathogenesis within the respiratory tract. Shotgun metatranscriptomic approaches may be particularly well suited to these types of analyses as they can survey both bacterial and non-bacterial microbes and may provide an assessment of functional activity.

#### Future perspectives: integrate the host response

Although technically challenging, high-throughput sequencing techniques, such as metatranscriptomics, offer the possibility to simultaneously sequence the host and microbial genomes.^36^ In principle, host genome and microbiome profiles could be analysed in ‘one run’ in airway samples from COPD patients, offering the possibility to directly analyse the interactions between microbiota and the host immune profiles on a personal genome level. Langelier *et al* recently demonstrated how the integration of host response and microbiome profiling can contribute to diagnosis and characterisation of LRT infections (LRTI) in critically ill patients with acute respiratory failure, based on metagenomic and metatranscriptomic sequencing in tracheal aspirate samples.^87^ Combining pathogen, microbiome and host gene expression metrics enhanced LRTI diagnosis in cases of unknown aetiology, showcasing the potential of this model to optimise diagnostics for airway diseases.

## Conclusion

In conclusion, high-throughput sequencing techniques allow for an increasingly detailed assessment of the COPD airway microbiome. Sputum likely only partially represents the respiratory microbiome in COPD, given the enrichment for oral flora. However, the increasing evidence of sputum microbiome associations with characteristics of stable COPD and exacerbations, treatment response, and outcomes suggest that sputum microbiome profiling may provide important biomarkers to better understand COPD biology and guide therapies. Many outstanding questions remain. Answering these questions will take exacting studies implementing standardised protocols and employing the evolving technologies and statistical approaches to study microbial community structure in relation to the COPD-relevant host response.
