# Supplementary material for: Sputum microbiome profiling in COPD: beyond singular pathogen detection
Source: Thorax. 2020 Jan 29;75(4):338–44. doi: 10.1136/thoraxjnl-2019-214168 (PMC7231454; doi:10.1136/thoraxjnl-2019-214168)
Supplement: Supplementary data [file thoraxjnl-2019-214168supp001.pdf]

Supplementary Table 1. Summary of studies investigating the microbiome in sputum samples of COPD participants

Author (Year)	Sequencing technique	Participants characteristics	Summary findings
Cabrera-Rubio, et al. (2012)[1]	16S rRNA	8 participants in stable phase	-Microbiome profiling in sputum, bronchial aspirate, BAL and bronchial mucosal biopsy revealed lower diversity in sputum samples compared to the other 3 sample types
Molyneaux, et al. (2013)[2]	16S rRNA, qPCR	14 participants in stable phase, 17 healthy controls	-After rhinovirus infection, there was a rise in bacterial burden and outgrowth of *Haemophilus influenza* from pre-existing microbiota in COPD participants. This was not observed in healthy controls.
Garcia-Nunez, et al. (2014)[3]	16S rRNA	17 participants in stable phase	-Most prevalent phyla were Proteobacteria, Firmicutes, and Actinobacteria<br>-Patients with moderate disease showed greater microbial diversity than patients with advanced disease<br>- Alpha diversity was decreased in patients with advanced disease
Galiana, et al. (2014)[4]	16S rRNA, qPCR	19 participants in stable phase	-Bacterial diversity was higher in patients with moderate COPD than with severe COPD<br>-Bacterial load was higher in severe COPD<br>-In severe COPD patients, the composition of bacterial genera differed more among themselves, than samples from the mild/moderate group
Huang, et al. (2014)[5]	16S rRNA, qPCR	12 participants; sample collection before, during and after AECOPD	-Abundance of several bacterial phyla differed between stable phase and AECOPD, where Proteobacteria abundance was increased during AECOPD<br>- Steroid and antibiotic therapy showed opposite effects on the microbiome, whereby steroid therapy increased Proteobacteria abundance<br>- *Haemophilus influenza* abundance was positively correlated to the abundance of phylogenetically related bacteria, whereas the abundance of other bacteria was negatively correlated
Barker, et al. (2015)[6]	16S rRNA, qPCR	120 participants in stable phase, 55 paired stable and AECOPD data	- *Haemophilus influenza* bacterial load was an independent predictor of sputum TNF-alpha and IL-1beta levels in stable state<br>- at AECOPD, change of *Moraxella catarrhalis* bacterial load compared to

			stable state correlated with change in sputum TNF-alpha and IL-1beta concentration; this was not observed in *Haemophilus influenza*
Millares, et al. (2015)[7]	16S rRNA, metagenomics	8 participants with severe COPD; sample collection during stable phase and AECOPD	- The relative abundance of bacterial phyla and genera were consistent between stable phase and AECOPD<br>-Functional pathways changed at AECOPD events, indicating a shift of the metabolic functionality of the microbiome towards AECOPD
Brill, et al. (2015)[8]	Sputum culture;16S rRNA, qPCR	99 participants in stable phase, 86 completed follow up,	-A randomized controlled trial investigating different antibiotic classes (moxifloxacin, doxycycline, azithromycin, placebo)<br>-Total airway bacterial load did not decrease after 3 months of antibiotic therapy<br>-Increases in antibiotic resistance in all treatment groups
Wang, et al. (2016)[9]	16S rRNA, qPCR	87 participants; sample collection during stable state, AECOPD, 2 weeks post-therapy and 6 weeks recovery	-Sputum microbiome profiles were dynamic from stable state towards AECOPD, involving the change of microbial diversities, abundances of bacterial communities and the outgrowth of “keystone bacteria”, such as *Haemophilus* or *Moraxella* spp.<br>-Microbiome structure and diversity were correlated with serum and sputum biomarkers<br>-Steroid and antibiotic therapy showed opposite effects on the microbiome with respect to diversity and outgrowth of individual bacteria and bacterial communities
Wang, et al. (2017)[10]	16S rRNA	281 participants; sample collection at baseline and AECOPD	- Microbiome composition shifted from highly diverse to less diverse during AECOPD, where few bacterial genera become predominant abundant<br>-Bacterial dysbiosis was associated with increased exacerbation severity, indicated by higher CAT scores and decrease of lung capacity<br>-Microbial dysbiosis, in concert with eosinophilic inflammation, was associated with even higher exacerbation severity
Leitao Filho, et al. (2018)[11]	16S rRNA	102 participants hospitalized due to AECOPD, followed for one year after discharge	- Microbiome profile in hospitalized AECOPD patients is significantly associated with 1-year mortality<br>-Reduced microbial diversity indicated poorer survival prognosis<br>-The combined absence of Veillonella/presence of Staphylococcus

			was associated with an increased risk of 1-year mortality by 85-fold
Ghebre, et al. (2018)[12]	16S rRNA, RT-PCR panel for common respiratory viruses	73 participants with AECOPD, 32 asthmatic patients with exacerbation	-Asthma and COPD patients share 3 exacerbation biological clusters after integrating microbiome profiles and host-inflammatory profiles<br>-Cluster 1: increased pro-inflammatory mediators, evidence of neutrophilic inflammation, bacteria-associated with increased proportions of Proteobacteria and Proteobacteria/Firmicutes ratio<br>-Cluster 2: increased blood and sputum eosinophils, type 2 mediators and increased proportions of Bacteroidetes<br>-Cluster 3: increased type 1 mediators and proportions of Actinobacteria and Firmicutes
Sinha, et al. (2018)[13]	16S rRNA	4 participants; sample collection during stable phase (two-days, 2-9 months)	-Alpha diversity is similar over a two-day period<br>-Microbiome variability was increased over a 9-months period<br>-Firmicutes was the most prevalent phylum, followed by Bacteroidetes
Mayhew et al. (2018)[14]	16S rRNA	101 participants, 584 (spontaneous and induced) sputum samples from stable and exacerbation time points over 1 year	-Subtypes of COPD have distinct bacterial compositions and stabilities over time<br>- Microbiome profiles show less variation within an individual than between individuals, however, some individuals exhibited high variability over time<br>- With increasing disease severity, the abundance of Proteobacteria increases, whereas diversity overall decreases<br>-No significant changes of diversity or taxa relative abundance between stable and exacerbation phase (with the exception of *Moraxella* spp.)<br>-COPD patients with higher exacerbation frequencies exhibit less stable lung microbiome over time<br>- Microbiome composition of bacterial exacerbations differs from viral and eosinophilic
Wang et al. (2019)[15]	16S rRNA, qPCR, host RNA microarray, Proteomic assay	16 healthy controls, 43 participants with COPD, sample collection during stable state, AECOPD, 2 weeks post-therapy and 6 weeks recovery, 6 months from	- stable COPD patients showed a significantly increased relative abundance of the genera *Moraxella*, *Streptococcus* and Actinobacteria, as well as decreased alpha diversity, compared to healthy controls<br>-the relative abundance of *Moraxella* was increased at stable state in GOLD III versus II patients and in ICS versus non-ICS exposed patients<br>- During AECOPD, the relative abundance of *Moraxella* was increased and alpha

		the first stable visit	diversity decreased compared to the stable state, along with significantly increased neutrophil and decreased macrophage percentage.<br>- the trend of increased *Moraxella* and decreased alpha diversity was reversed at post- exacerbation time points<br>- significant associations between *Moraxella* and *Haemophilus* with host transcriptome and proteome profiles of host interferon and pro-inflammatory signaling pathways and neutrophilic inflammation

qPCR=quantitative PCR; AECOPD= acute exacerbations of COPD

REFERENCES:

1. Cabrera-Rubio R, Garcia-Núñez M, Setó L, et al. Microbiome diversity in the bronchial tracts of patients with chronic obstructive pulmonary disease. *J Clin Microbiol*. 2012;50(11):3562-3568.

2. Molyneaux PL, Mallia P, Cox MJ, et al. Outgrowth of the bacterial airway microbiome after rhinovirus exacerbation of chronic obstructive pulmonary disease. *Am J Respir Crit Care Med*. 2013;188(10):1224-1231.

3. Garcia-Nunez M, Millares L, Pomares X. Severity-Related Changes of Bronchial Microbiome in Chronic Obstructive Pulmonary Disease. *J Clin Microbiol*. 2014;52(12):4217-4223.

4. Galiana A, Aguirre E, Rodriguez JC. Sputum microbiota in moderate versus severe patients with COPD. *Eur Respir J*. 2014;43(6):1787-1790.

5. Huang YJ, Sethi S, Murphy T, et al. Airway microbiome dynamics in exacerbations of chronic obstructive pulmonary disease. *J Clin Microbiol*. 2014;52(8):2813-2823.

6. Barker BL, Haldar K, Patel H, et al. Association between pathogens detected using quantitative polymerase chain reaction with airway inflammation in COPD at stable state and exacerbations. *Chest*. 2015;147(1):46-55.

7. Millares L, Pérez-Brocal V, Ferrari R, et al. Functional metagenomics of the bronchial microbiome in COPD. *PLoS One*. 2015;10(12): e0144448.

8. Brill SE, Law M, El-Emir E, et al. Effects of different antibiotic classes on airway bacteria in stable COPD using culture and molecular techniques: A randomised controlled trial. *Thorax*. 2015;70(10):930-938.

9. Wang Z, Bafadhel M, Haldar K, et al. Lung microbiome dynamics in COPD exacerbations. *Eur Respir J*. 2016;47(4):1082-1092.

10. Wang Z, Bafadhel M, Haldar K, et al. Lung microbiome dynamics in COPD exacerbations. *Eur Respir J*. 2016;47(4):1082-1092.

11. Leitao Filho FS, Alotaibi NM, Ngan D, et al. Sputum microbiome is associated with 1-

year mortality after chronic obstructive pulmonary disease hospitalizations. *Am J Respir Crit Care Med* 2019;199:1205–1213.

12. Ghebre MA, Pang PH, Diver S, et al. Biological exacerbation clusters demonstrate asthma and chronic obstructive pulmonary disease overlap with distinct mediator and microbiome profiles. *J Allergy Clin Immunol*. 2018;141(6):2027-2036.e12.

13. Sinha R, Weissenburger-Moser LA, Clarke JL, et al. Short term dynamics of the sputum microbiome among COPD patients. *PLoS One*. 2018;13(3):e0191499.

14. Mayhew D, Devos N, Lambert C, et al. Longitudinal profiling of the lung microbiome in the AERIS study demonstrates repeatability of bacterial and eosinophilic COPD exacerbations. *Thorax*. 2018;73(5):422-430.

15. Wang Z, Maschera B, Lea S, et al. Airway host-microbiome interactions in chronic obstructive pulmonary disease. *Respir Res*. 2019;20(1): 113.
